# Endovascular Treatment of Gastrointestinal Hemorrhage

**DOI:** 10.3390/medicina58030424

**Published:** 2022-03-14

**Authors:** Martin Vorčák, Ján Sýkora, Martin Ďuríček, Peter Bánovčin, Marián Grendár, Kamil Zeleňák

**Affiliations:** 1 Clinic of Radiology, Jessenius Faculty of Medicine, Comenius University in Bratislava, Malá Hora 10701/4A, 03601 Martin, Slovakia; martin.vorcak@gmail.com (M.V.); jan.sykora@uniba.sk (J.S.); 2 Clinic of Radiology, University Hospital in Martin, Kollárova 2, 03659 Martin, Slovakia; 3 Clinic of Gastroenterological Internal Medicine, Jessenius Faculty of Medicine, Comenius University in Bratislava, Malá Hora 10701/4A, 03601 Martin, Slovakia; martin.duricek@uniba.sk (M.Ď.); peter.banovcin2@uniba.sk (P.B.); 4 Clinic of Gastroenterological Internal Medicine, University Hospital in Martin, Kollárova 2, 03659 Martin, Slovakia; 5 Bioinformatic Center, Jessenius Faculty of Medicine, Comenius University in Bratislava, Malá Hora 10701/4A, 03601 Martin, Slovakia; marian.grendar@uniba.sk

**Keywords:** gastrointestinal bleeding, embolization, angiography

## Abstract

*Background and Objectives*: Severe non-variceal gastrointestinal bleeding is a life-threatening condition with complicated treatment if endoscopic therapy fails. In such cases, transcatheter arterial embolization is recommended. The technical and clinical effects of this technique were analyzed in this group of patients, as well as its complication rate and 30-day mortality. *Materials and Methods*: Patient data over a one-decade period (from 2010 to 2019) were analyzed retrospectively; 27 patients (18 men and 9 women; median age 61 years) treated by endovascular embolization in our institution, with clinically significant gastrointestinal hemorrhage after unsuccessful or impossible endoscopic treatment, were identified, and their data were collected. *Results*: The source of bleeding was found in 88% of patients, but embolization was performed in 96% of them. The overall technical success rate was 96.8%, and the clinical success was 88.5%. Re-bleeding occurred in eight cases, five of whom had re-embolization that was technically successful in four cases. The incidence of re-bleeding was significantly higher in patients with two or more comorbidities (*p* = 0.043). There was one serious complication (4%) in the group, and minor difficulties occurred in 18% of patients; 30-day mortality reached 22%. Mortality was significantly higher in the group of patients with re-bleeding (*p* = 0.044). *Conclusions*: Transcatheter arterial embolization is a mini-invasive method with high technical success in patients with endoscopically untreatable gastrointestinal bleeding; it is also suitable for high-risk cases. Mortality (to a significant extent) depends on the occurrence of re-bleeding and the patient’s comorbidities.

## 1. Introduction

Non-variceal gastrointestinal tract (GIT) bleeding is often a sudden, life-threatening condition. In 85% of patients, we encounter bleeding from the upper GIT [1], which includes the part of the GIT from the esophagus to the ligament of Treitz, and lower GIT bleeding, which includes bleeding from the small intestine, colon, and rectum. Most bleeding episodes resolve spontaneously or after conservative treatment. Endoscopy is the method of choice for the diagnosis and treatment of GIT bleeding. Nevertheless, there is a group of 5–10% of patients in whom it is not possible to achieve hemodynamic stability, or endoscopy is ineffective or unfeasible after surgery. These patients require endovascular or surgical intervention [2]. Technical improvement of transcatheter arterial embolization (TAE), based on the possibility of using microcatheters and new embolic agents, enables targeted superselective embolization, with high technical and clinical success. The aim of our study was to evaluate the technical and clinical success of endovascular treatment of gastrointestinal bleeding, as well as the complication rate and 30-day mortality.

## 2. Materials and Methods

Retrospective analysis of patients with clinically significant GIT bleeding (nonresponsive to medical, endoscopic or, in some cases, surgical treatment) who underwent angiography and embolization was performed in the local database of Clinic of Radiology in the period from 2010 to 2019.

The procedures were performed on a Siemens Axiom Atlas Monoplane angiograph. A transfemoral approach with a 6F introducer (Super Sheath 25 cm; Boston Scientific, Cork, Ireland) was used to perform splanchnic angiography. Once, in a patient with an advanced rectal tumor (TU), the approach was changed to transaxillary, due to the technical unavailability of the inferior mesenteric artery. For embolization, a coaxial system with a guiding catheter was used (55 cm RDC Vista bride tip, Cordis, FL, USA; or 100 cm Sim 2 Envoy, Cerenovus, Le Locle, Switzerland). Embolization was performed selectively through a microcatheter (based on the needed size: Direxion 0.021 in × 130 cm, Boston Scientific, Cork, Ireland; or Excelsior SL-10 0.0165 in × 150 cm, Stryker Neurovascular, Cork, Ireland). Coils (Target, Stryker Neurovascular, Cork, Ireland), liquid embolic agent (LEA–Onyx 18, Covidien, Plymouth, MN, USA; or Phil 25%; MicroVention, CA, USA), and microparticles (different-sized Embospheres, Biosphere Medical, Roissy, France) or Spongostan foam (Gelita Medical, Eberbach, Germany) were used as embolic materials. To achieve the optimal position, the microcatheter was navigated using the following guidelines according to the operator’s preference (Asahi Meister 16, 180 cm, DA, Asahi, Aichi, Japan; or Hybrid Wire 007J, 12-14DA; Balt Extrusion, Montmorency, France). In selected patients with negative angiographic findings, empirical embolization was performed, or embolization was oriented via an endoscopically placed clip close to the area of the bleeding lesion.

Technical success was determined as the angiographic disappearance of extravasation or the occlusion of pseudoaneurysm or other embolized vascular pathology at the end of embolization. Clinical success was defined as the disappearance of the original symptoms of bleeding after the endovascular procedure. Recurrent bleeding was defined as a repeatedly significant decrease in hemoglobin (Hb) after embolization over the period of monthly follow-up. Complications were classified as serious if they caused a prolongation in hospital stay or required surgical treatment, or minor if they did not meet the criteria above.

Data were analyzed using Fisher’s exact test and the Wilcoxon signed-rank test. Statistical significance was determined at *p* = 0.05. Statistical software R, version 4.0.2, was used for data processing [3].

## 3. Results

In total, 27 patients (18 men and 9 women) with a median age of 61 years (range 2–94, IQR 53–76) met the inclusion criteria. One-third of the patients were older than 70 years. Overall, 78% of patients had a severe comorbidity, and 55% were polymorbid ill patients with two or more comorbidities. The demographic data, comorbidities, and clinical presentation of bleeding are shown in detail in Table 1.

**Figure 1:**
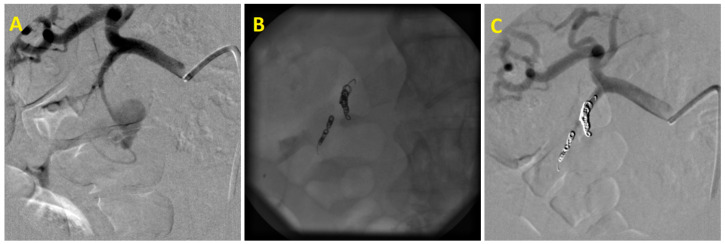
Embolization of superior anterior pancreaticoduodenal artery pseudoaneurysm: (**A**)—Superior anterior pancreaticoduodenal artery pseudoaneurysm after pancreatic pseudocyst endoscopic treatment. (**B**)—Embolic agent–coils. (**C**)—Final angiogram confirming pseudoaneurysm occlusion.

The median time interval between initial clinical manifestations and angiography was 5.5 days (14 h to 22 days). Pathological angiographic findings were confirmed in 24 out of 27 patients (88%). Active bleeding was identified in nine cases, pseudoaneurysms in seven patients (Figure 1), tumor enhancement was found four times, and pathological hypervascularization in the ulcer area was observed in two cases. Both arterioportal fistula and pseudoaneurysm with arteriovenous fistula in the liver were found once each. In three cases, it was not possible to angiographically identify the source of hemorrhage. Despite negative angiographic findings, two of these patients also underwent empirical embolization of the gastroduodenal artery, with continued bleeding from the duodenal ulcer, which was initially treated endoscopically. In total, embolization was performed in 26 patients (96%). The etiology of the hemorrhage, treated arteries, and used embolic materials are shown in Table 2.

The technical success rate of embolization in our group of patients reached 100%. Clinical success or cessation of clinical signs after embolization reached 88.5% (23/26). Recurrent bleeding occurred in eight patients (29.6%). In five cases, repeat embolization was performed, which was technically and clinically successful in four patients. After including interventions for re-bleeding, technical success was achieved in 31/32 interventions (96.8%). The characteristics of patients with re-bleeding are summarized in Table 3. 

A significantly higher risk of re-bleeding occurred in polymorbid patients (*p* = 0.043). In patients with re-bleeding, there is a tendency, although insignificant, of patients being of older age, with a median age of 70 (SD 63–76) years, compared to patients without re-bleeding, with a median age of 58 (SD 35–68) years (*p* = 0.067).

There was only one major procedure-related complication in the patient group (4%): splenic necrosis that developed into abscess occurred in one patient after splenic artery embolization with coils and LEA, because of massive arterial bleeding from this artery. This complication was managed by surgical splenectomy. Other complications, classified as minor, were present in a total of five patients (18.5%)—three periprocedural dissections of the artery without hemodynamic effects or clinical manifestation, one ulceration of the gastric mucosa after embolization of the left gastric artery, and one case of puncture site bleeding managed by prolonged compression and a hemostatic bandage.

The 30-day mortality reached 22% (six patients). The median age of selected patients who died was 68 years vs. 58 years in the surviving patients. The 30-day mortality was significantly increased by the presence of re-bleeding (11% vs. 50%; *p* = 0.044). Two critical patients died from persistent bleeding: a polymorbid 94-year-old patient with a duodenal ulcer bleeding, and a 67-year-old patient with inoperable pancreatic cancer. Deaths in the group of patients without persistent bleeding were caused by multiorgan failure due to malignancy (two cases), myocardial infarction (one case), and hepatorenal failure (one case).

## 4. Discussion

Upper GIT bleeding is more common, accounting for approximately 70% of GIT bleeds [4], with ulcer disease being the most common cause [5]. In hemodynamically unstable patients with upper gastrointestinal bleeding, intensive care is needed for patient stabilization, followed by subsequent endoscopic examination, which has a high therapeutic success rate in this location. In contrast, hemodynamically significant lower GIT bleeding is less common. Hemodynamic instability and inadequate bowel cleansing are considered among the relative contraindications to colonoscopy. In the acute phase, colonoscopy can localize the source of bleeding in only 42% of cases [6]. A CT scan performed with a proper protocol is able to detect GIT bleeding with a higher sensitivity compared to that in conventional angiography (0.3 mL/min vs. 0.5 mL/min) [4]. CT examination is therefore recommended before digital subtraction angiography (DSA) in cases of bleeding from the lower GIT and endoscopically non-localized bleeding from the upper GIT; its implementation in cases of endoscopically localized and untreatable upper GIT bleeding remains questionable, when endoscopy should provide sufficient information to perform DSA [7].

Indications for endovascular treatment include technical failure of endoscopic treatment, recurrent bleeding despite a second endoscopic treatment and an endoscopically non-localizable source of bleeding [4,8]. Contraindications to standard angiographic examination are only relative contraindications in life-threatening bleeding.

The development of embolic materials has provided various options for use according to the desired properties and nature of embolization. Coils, alone or in combination with Spongostan, have been used in GIT bleeding [7,9,10]. The use of coils or Spongostan as the sole embolic material is associated with an increased risk of recurrent bleeding [2,11]. An advantage of LEAs is immediate hemostasis, which is especially needed in hemodynamically unstable patients and patients with coagulopathy; they have proven successful in the treatment of GIT bleeding [12,13,14,15,16,17]. Microparticles are frequently used in the embolization of bleeding tumors; for intestinal embolization, a size larger than 500 µm is recommended. Ischemic complications are uncommon in the upper GIT area thanks to the extensive collateral network. Embolization is associated with a higher risk of ischemic complications in the lower GIT. Due to superselective embolization and occlusion of less than three vasa recta, this risk is minimal [18].

Empirical or blind embolization refers to embolization based on endoscopic findings without confirmed extravasation during angiography; it is accepted in cases of upper GIT bleeding, and published results do not differ from embolization of proven hemorrhage [19]. This technique was performed by our team in two cases of refractory bleeding duodenal ulcer, with technical and clinical success.

**Figure 2:**
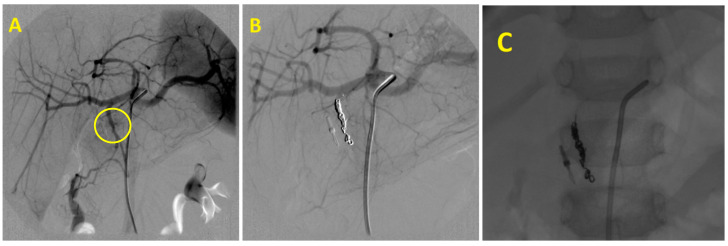
Gastroduodenal artery embolization in a patient with endoscopically untreatable duodenal ulcer bleeding: (**A**)—Duodenal wall hypervascularity (yellow circle). (**B**)—The final angiogram with the occlusion of the gastroduodenal artery. (**C**)—Embolic material–coils positioned next to the endoscopically placed clip, facilitating endovascular treatment and precise targeting.

The embolization procedure can be facilitated by an endoscopically placed clip on the edge of a suspicious lesion. This helps the radiologist to target the local treatment of the bleeding area (Figure 2). Recent findings suggest the potential benefits of preventive embolization in bleeding duodenal ulcers in patients at significant risk of re-bleeding [20].

In a published analysis that included 15 studies (a total of 829 patients) focusing on TAE in upper GIT bleeding, the technical success rate was 93% (62–100%), the clinical success rate was 67% (52–94%), the risk of re-bleeding was 33% (9–66%), and the 30-day mortality was 28% (4–46%) [21]. The results of embolization from the lower GIT showed a technical success rate above 90%, a clinical favorable outcome rate of 86%, and an occurrence of ischemic complications of 4–6% [22,23]. Outcomes can be compared to presented series in Table 4.

Our study has limitations; among them, the retrospective and single-center design is the most relevant. Other limitations include the sample being small in volume and the wide range of embolized pathology. However, we present real experience from one center, where all pathological lesions detected by angiography were treated. Notable clinical success with an acceptable re-bleeding rate of 29.6% was confirmed by our analysis. Importantly the re-bleeding rate depended significantly on the presence of comorbidities. In addition to the number of comorbidities, other studies have identified additional risk factors for early recurrent hemorrhage, including coagulopathy, prolonged time from bleeding to angiography, and a higher number of transfusions [21]. The total 30-day mortality was 22%, and it was significantly higher in the group of patients with recurrent bleeding.

## 5. Conclusions

Transcatheter embolization is the recommended treatment option in the case of endoscopically untreatable gastrointestinal bleeding; it has a high technical success rate and an acceptable level of complications, and is also suitable for high-risk patients, whose mortality mostly depends on the occurrence of early recurrent hemorrhage and the presence of comorbidities.

## Figures and Tables

**Table 1:** Patient demographics.

Variables	*n* = 27
Gender M/F (*n*)	18/9
Age (median, IQR)	61 (53–76)
Pre-procedural Hb (g/L) (median, IQR)	88 (78–96)
Comorbidities:	*n* (%)
Malignancy	13 (48.1)
HT	15 (55.5)
CAD	7 (25.9)
Heart failure	1 (3.7)
Arrhythmia	2 (7.4)
Respiratory failure	1 (3.7)
Severe DM	3 (11.1)
PAD	0
Cirrhosis	3 (11.1)
Coagulopathy	4 (14.8)
Clinical presentation	*n* (%)
Enterorrhagia	8 (30)
Hematemesis	6 (22)
Melena	4 (15)
Hematemesis with melena	5 (18)
Enterorrhagia followed by melena	4 (15)

M—male, F—female, IQR—interquartile range, Hb—hemoglobin, HT—hypertension, CAD—coronary artery disease, DM—diabetes mellitus, PAD—peripheral arterial disease.

**Table 2:** Etiology of gastrointestinal bleeding, treated arteries, and used embolic materials.

Variables	*n* (%)
Etiology:	
Duodenal ulcer	5/27 (18.5)
Malignancy	7/27 (25.9)
Iatrogenic:	
Endoscopic drainage	2/27 (7.4)
Surgery	7/27 (25.9)
Pancreatic pseudoaneurysm	3/27 (11.1)
Jejunal dysplasia	2/27 (7.4)
Mallory–Weiss syndrome	1/27 (3.7)
Embolized arteries:	
Gastroduodenal artery	4/26 (15.4)
Left gastric artery	4/26 (15.4)
Superior pancreaticoduodenal artery	3/26 (11.5)
Inferior pancreaticoduodenal artery	3/26 (11.5)
Hepatic artery	3/26 (11.5)
Rectal artery	3/26 (11.5)
SMA jejunal branches	3/26 (11.5)
Splenic artery	2/26 (7.7)
Great pancreatic artery	1/26 (3.8)
Embolic materials:	
Coils	15/26 (57.7)
LEA	5/26 (19.2)
Microparticles	2/26 (7.7)
Spongostan	1/26 (3.8)
Coils + microparticles	1/26 (3.8)
Coils + LEA	1/26 (3.8)
Microparticles + LEA	1/26 (3.8)

SMA—superior mesenteric artery, LEA—liquid embolic agent.

**Table 3:** Characteristics of patients with recurrent bleeding.

Patient	Time to Re-Bleed (Days)	Primary Diagnosis	Primary Endovascular Treatment	Secondary Treatment	Technical Success	Clinical Success	30-Day Mortality
No. 2	3	Rectal cancer	Spongostan	TAE-LEA	Successful	Successful	
No. 4	3	NET	Coils	Surgical revision, conservative treatment	-	Successful	
No. 7	11	Jejunal angiodysplasia	Coils	Conservative treatment	-	Successful	
No. 9	3	Post-surgery Klatskin TU	Coils	TAE-LEA	Successful	Successful	Death
No. 10	10	Post-surgery pancreatic cancer	No pathology revealed, no treatment	TAE-Coils and LEA	Successful	Successful	
No. 11	1	Duodenal ulcer	Coils	TAE technically unsuccessful	Unsuccessful	Unsuccessful	Death
No. 17	9	Gastric cancer	Coils and particles	TAE–coils	Successful	Successful	Death
No. 27	17	Pancreatic cancer	LEA	Conservative treatment	-	Unsuccessful	Death

LEA—liquid embolic agent, NET—neuroendocrine tumor, TAE—transcatheter arterial embolization, TU—tumor.

**Table 4:** Results of presented series.

Results	*n* (%)
Overall technical success	31/32 (96.8)
Clinical success	23/26 (88.5)
Re-bleeding	8/26 (29.6)
Complications:	
Major	1/26 (4)
Minor	5/26 (18.5)
30-day mortality	6/26 (22)

## Data Availability

The datasets used and analyzed in the current study are available from the corresponding author upon reasonable request.
